# The adaptation of a CTN-1 rabies virus strain to high-titered growth in chick embryo cells for vaccine development

**DOI:** 10.1186/1743-422X-11-85

**Published:** 2014-05-12

**Authors:** Caiping Guo, Chunhua Wang, Shan Luo, Shimao Zhu, Hui Li, Yongdi Liu, Lanzhen Zhou, Pei Zhang, Xin Zhang, Yujiang Ding, Weirong Huang, Kaiyong Wu, Yanpeng Zhang, Weihua Rong, Hua Tian

**Affiliations:** 1 Shenzhen Weiguang Biological Products Co., Ltd, Shenzhen 518107, Guangdong province, China

**Keywords:** Rabies virus, CTN-1 V, Chick embryo cells, Adaptation, Vaccine

## Abstract

**Background:**

Rabies virus is the causative agent of rabies, a central nervous system disease that is almost invariably fatal. Currently vaccination is the most effective strategy for preventing rabies, and vaccines are most commonly produced from cultured cells. Although the vaccine strains employed in China include CTN, aG, PM and PV, there are no reports of strains that are adapted to primary chick embryo cells for use in human rabies prevention in China.

**Results:**

Rabies virus strain CTN-1 V was adapted to chick embryo cells by serial passage to obtain the CTNCEC25 strain. A virus growth curve demonstrated that the CTNCEC25 strain achieved high titers in chick embryo cells and was nonpathogenic to adult mice by intracerebral inoculation. A comparison of the structural protein genes of the CTNCEC25 strain and the CTN-1 V strain identified eight amino acid changes in the mature M, G and L proteins. The immunogenicity of the CTNCEC25 strain increased with the adaptation process in chick embryo cells and conferred high protective efficacy. The inactivated vaccine induced high antibody responses and provided full protection from an intramuscular challenge in adult mice.

**Conclusions:**

This is the first description of a CTNCEC25 strain that was highly adapted to chick embryo cells, and both its in vitro and in vivo biological properties were characterized. Given the high immunogenicity and good propagation characteristics of the CTNCEC25 strain, it has excellent potential to be a candidate for development into a human rabies vaccine with high safety and quality characteristics for controlling rabies in China.

## Background

Rabies is a widespread neurological disease and is an ancient fatal encephalitis with nearly 100% mortality. Rabies reportedly causes approximately 55,000 human deaths annually throughout the world, the majority of which occur in Asia [1]. Following India, China has the second highest number of human cases in the world [2]. The causative agents of rabies are viruses belonging to the *Lyssavirus* genus in the family *Rhabdoviridae* of which the prototypic rabies virus (RABV) is responsible for the vast majority of cases. The RABV genome is a single-stranded, negative-sense RNA of approximately 12 kb encoding five structural proteins, and its order (3’ to 5’) is nucleoprotein (N), phosphoprotein (P), matrix protein (M), glycoprotein (G) and RNA-dependent RNA polymerase (L) [3]. Between each of the five structural genes are four non-transcribed intergenic regions of different lengths. In addition, there are two non-coding regions at the end of the genome, namely the 3’ leader and the 5’ trailer, which are involved in regulating viral gene transcription and genome replication [4].

At present, vaccination is the most effective method to prevent rabies and the development of human rabies vaccines follows a trend from brain passage to cell adaptation primarily because of safety considerations [5]. Rabies vaccines have improved greatly since 1885 when Louis Pasteur successfully vaccinated a boy who was bitten by a rabid dog, using the spinal cord of a rabbit that had died of rabies as a vaccine for the first time. Phenol was then employed to treat Pasteur’s vaccine, not only for improved safety but also as a preservative to prevent bacterial contamination [6,7]. To minimize the adverse effects associated with nerve tissue vaccines caused mainly by myelin in these preparations, avian embryos and neonatal rodent brains were used to prepare the human vaccine. However, although it was relatively safer compared with nerve tissue vaccines, significant poor antigenic responses and severe adverse reactions were reported with embryo-derived rabies tissue vaccines [8]. The advent of cell culture vaccines has greatly improved the capacity for producing high quality vaccines. The first tissue culture rabies vaccine was derived from a virus grown in primary hamster kidney cells in the 1960s, followed by the human diploid cell vaccine (HDCV) in the mid-1970s [9,10]*.* An alternative to HDCV was the use of purified chick embryo cells (PCEC) [10] and vaccines produced from the Vero continuous cell lines [11]. During the past two decades, there have been numerous attempts to develop alternatives. The ability to clone the RABV G protein into bacterial plasmids and then express the protein in a range of systems has led to a number of alternative approaches with the potential for new vaccines against rabies [12-17]. However, because of the cost and challenge of gaining vaccine acceptance, cell culture vaccines will still rank as the most commonly used human rabies vaccines in the future [10].

Today, HDCV is the gold standard of rabies vaccines, but its high cost limits its use around the world, especially in developing countries. Alternatively, the PCECV, which is prepared from a fixed RABV strain grown in primary cultures of chicken fibroblast cultures, and it is much cheaper and has a similar safety and potency compared to that of HDVC. Therefore, the PCECV is a more advisable choice for human inoculation. However, because CEC-adapted RABV strains were not available, no PCECV has been used for rabies prevention in China. In this study, we describe a highly chick embryo cells (CECs) adapted RABV strain derived from a China fixed vaccine CTN-1 strain called CTNCEC25, and we investigate its biological properties in vivo and in vitro. Given the high immunogenicity and good propagation characteristics of the CTNCEC25 strain, it has excellent potential for development into an inactivated vaccine for human use. To the best of our knowledge, this is the first report of a CTN-1 strain that has been adapted to CECs and characterized systemically.

## Results

### Viral titers

**Figure 1:**
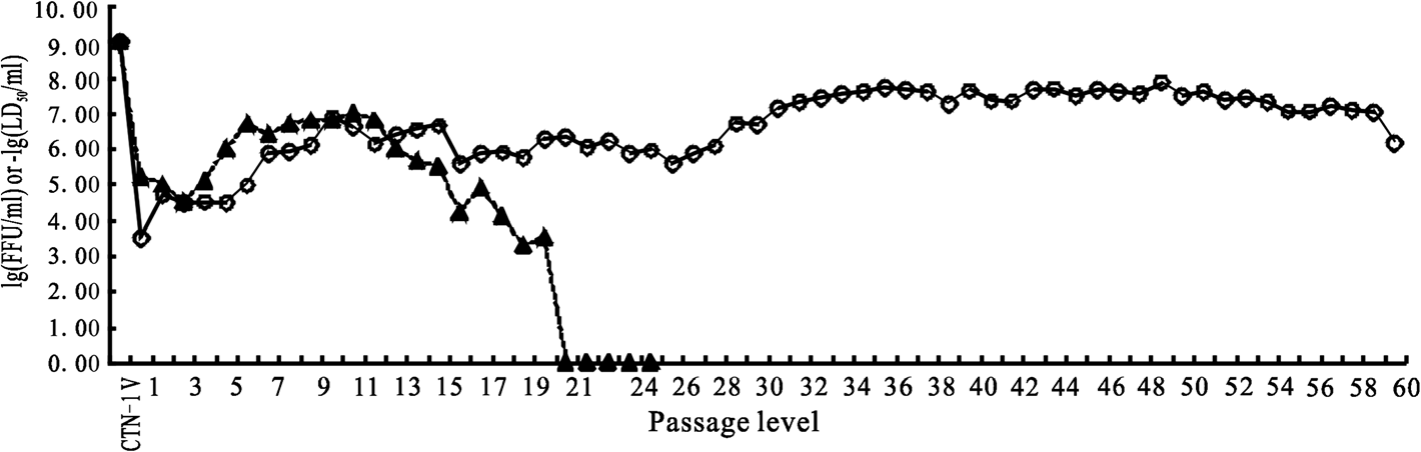
**Titers and LD**_**50 **_**change of the virus after passage in CECs.** The CTN-1 V strain was propagated in Vero cells and then transferred to CECs. The replication fitness of the virus in cultured cells or in adult mice were investigated using the fluorescence focus inhibition test (circle solid line) and the MIT test (black triangle dotted line), respectively.

To investigate the virus propagation properties during their passage in cultured cells, each virus passage was investigated. With serial passages in Vero cells, the titer of the CTN-1 V strain initially increased rapidly, reaching 10^9.0^ FFU/ml at passage 15 (Figure 1). However, initially, when transferred to the chick embryo and CECs, the viruses propagated poorly on the cells. The virus titers initially dropped sharply to a low of 10^4.5^ FFU/ml in the chick embryos and the first four CEC passages. Thereafter, the settling increased gradually over the course of the passages, and the viral titers increased from passage 5 up to 10^7.3^ FFU/ml at passage 31. The virus titers then reached a plateau at 10^7.3^-10^7.9^ FFU/ml between passages 32 and 57, indicating that the CTN-1 V strain was adapted to grow in CECs, and this virus strain was renamed the CTNCEC25 strain. However, further passage in CECs induced a slight decrease in the virus titer, and the virus only reached 10^6.2^ FFU/ml at passage 60 (Figure 1).

We also performed the MIT test to monitor the virulence of different virus passages in adult mice. As shown in Figure 1, the virulence of the virus dropped greatly to approximately 10^-5.0^ FFU/ml in the chick embryo passage and at the early CEC passages from 10^-9.0^ FFU/ml of the parental CTN-1 V, which was correlated with that of the fluorescence focus inhibition test. However, although the virus titers exhibited a stable increase during viral replication in the CECs from passages 4 to 10, the viral virulence in adult mice declined rapidly and was apathogenic at passage 20, although the virus titers in the cultured cells were maintained at approximately 10^6.0^ FFU/ml. These data indicated that the virus completely lost its lethality in adult mice during its adaptation to CEC.

### Pathogenicity study

**Figure 2:**
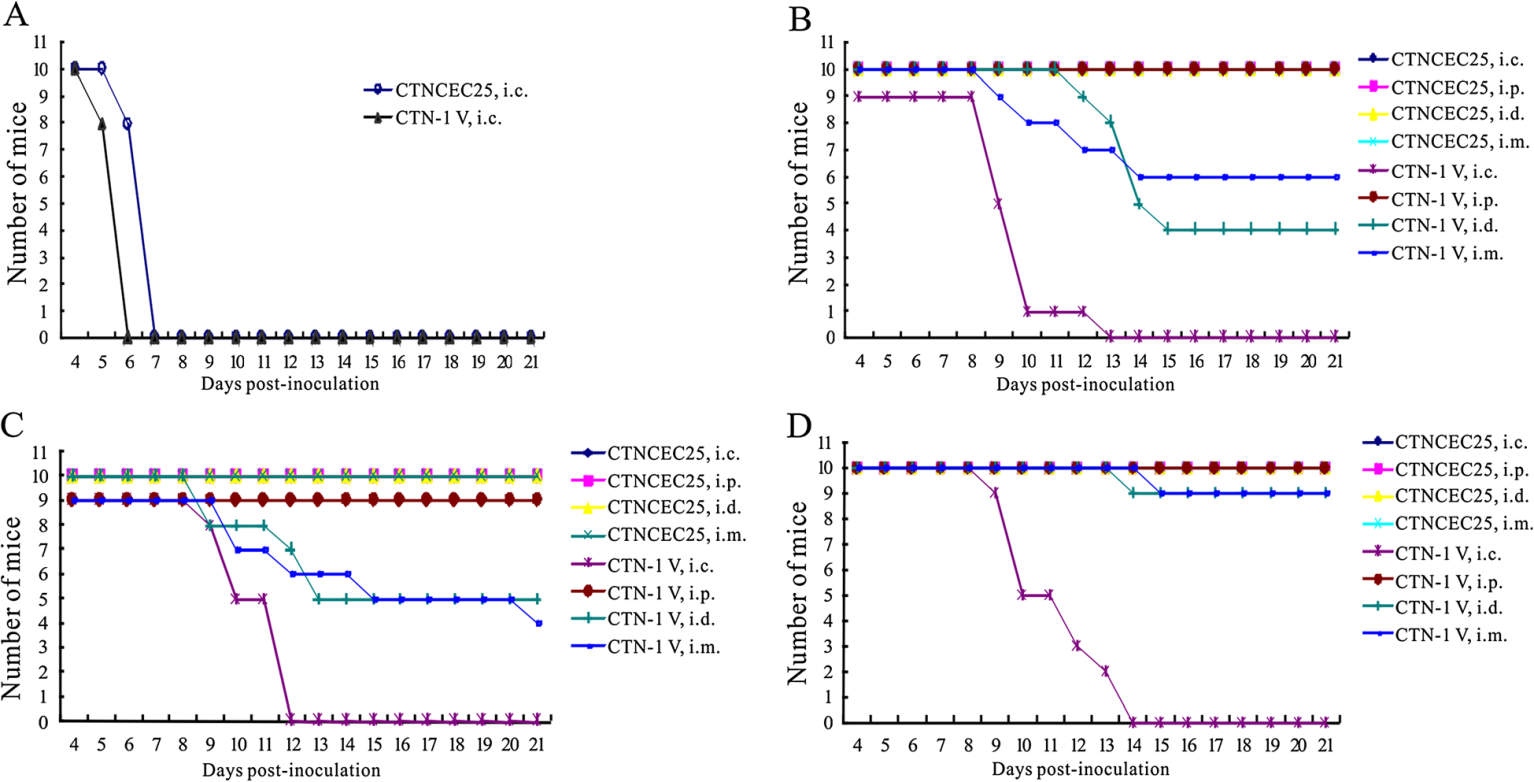
**Survival of mice inoculated with CTN-1 V and CTNCEC25. A**. Mortality of suckling mice injected i.c. with the CTN-1 V and CTNCEC25 strains. Mortality of 11–13 g **(B)**, 18–20 g **(C)** or 28–30 g **(D)** adult mice injected i.c., i.p., i.d. and i.m. with the CTN-1 V or CTNCEC25 strain. A group of ten two-day-old Kunming suckling mice or adult mice of different sizes were inoculated with the CTNCEC25 and CTN-1 V strains, respectively. The clinical disease signs and death number were observed and scored daily for 21 days. The production of anti-rabies antibodies in surviving mice was evaluated at the end of the experiment.

The MIT results shown above indicated that the CTNCEC25 strain lost its lethality to adult mice. To further determine the characteristics of the CTNCEC25 strain, a pathogenicity comparison study between the CTNCEC25 (passage 36) and CTN-1 V strains in mice at different ages was performed (Figure 2). The suckling mice inoculated i.c. with CTN-1 V showed rabies signs at day 4 pi, and all of the mice died at day 6 pi. As for the CTNCEC25 strain, signs were observed at day 5 pi, and all of the mice died on day 7 pi. Therefore, both the parental CTN-1 V strain and the CTNCEC25 strain caused complete mortality in suckling mice, although a one-day delay in the onset of disease and death was noted in mice inoculated with CTNCEC25 compared with the CTN-1 V strain (Figure 2A).

Conversely, although the CTN-1 V strain caused no deaths when injected intraperitoneally (i.p.) into adult mice, it elicited 100% mice mortality by intracerebral (i.c.) inoculation, and the mortality rates ranged from 60% to 10% when injected intradermally (i.d.) or intramuscularly (i.m.), depending on the mouse age, weight and administrative route (Figure 2B-D). In contrast, the CTNCEC25 strain caused no mortality regardless of the mouse age and weight and the administration route. In addition, all of the surviving mice inoculated with both viruses revealed no signs of rabies such as weight reduction and hyperactivity (data not shown), and the antibody titers from their serum ranged from 4.10 IU/ml to 33.99 IU/ml. Therefore, the above results showed that the CTNCEC25 strain was apathogenic to adult mice.

### Immunogenicity

**Figure 3:**
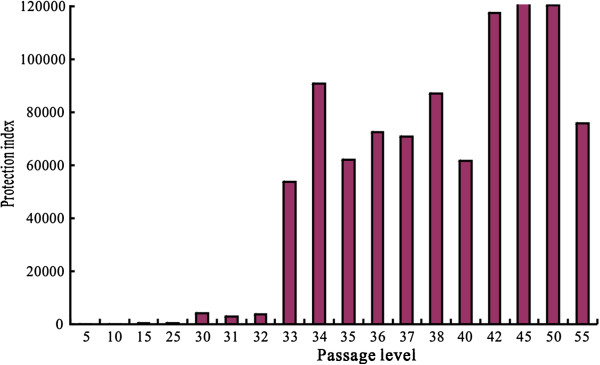
**The immunological efficacy of different viral passages during adaptation to CECs.** Adult mice were injected i.p. with vaccine candidates or PBS as a negative control. At 14 days after the first immunization, the adult mice were challenged by injecting i.c. with serial ten-fold dilutions of standardized viruses of the CVS strain. The numbers of mice dying of rabies between 4 and 14 days post-challenge were recorded, and the protection index was determined as the LD_50_ ratio of the experimental group to the LD_50_ of the control group.

The immunological efficacy of the CTNCEC25 strain was determined during passage in CECs. As shown in Figure 3, the immunological efficacy of the CTNCEC25 strain increased with the adaptation process in CECs. The protection index grew > 100 after 15 passage levels, >1,000 after 30 passage levels, >100,000 after 33 passage levels, and it reached the highest plateau of 128,825 at passage 45. The high protection efficacy of the CTNCEC25 strain was maintained from passage 33 to 55.

**Figure 4:**
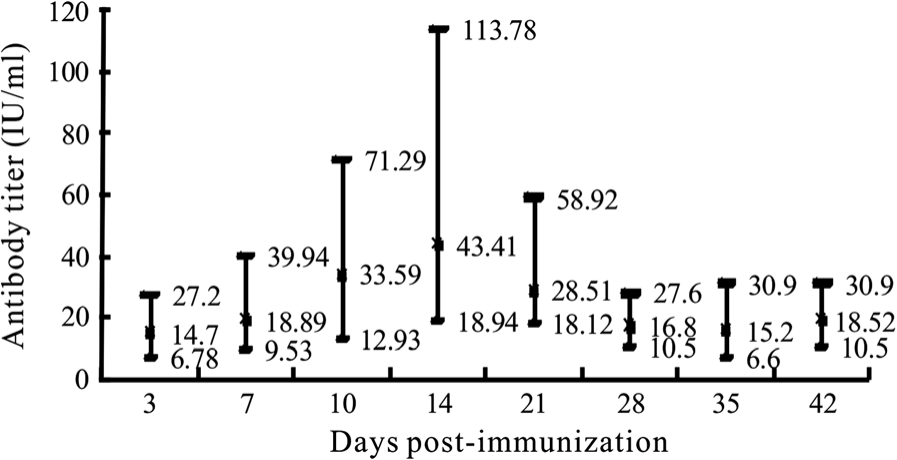
**The anti-rabies antibody levels of mice immunized with virus prepared from the CTNCEC25 strain (passage 36).** A group of five adult mice was used for each day point, and the potency of the prepared vaccine was determined according to the NIH test. The highest, mean and lowest results are indicated.

The results of the NIH test illustrated that vaccines prepared using the CTNCEC25 viral strain (passage 36, 40, and 45) as a seed had a potency equal to 6.65 IU/ml, 6.45 IU/ml and 4.01 IU/ml, respectively, which were all greater than the WHO-recommended standard of 2.5 IU/ml. The anti-rabies antibodies of mice immunized with virus prepared from passage 36 is shown in Figure 4. Mice inoculated with the vaccine produced high detectable antibody titres (mean titer: 14.7 IU/ml) as early as 3 days pi. The antibody titers rose quickly and reached a plateau with an average titer of 43.41 IU/ml (ranging from 18.94 to 113.78 IU/ml) at 14 days pi. After the antibody titers plateaued, the antibodies decreased gradually to a relatively low level but were constantly above 2.5 IU/ml.

These results indicated clearly that the CTNCEC25 strain could induce a strong protective immune response in animals and that the CTNCEC25 strain has potential for use as a viral seed for inactivated vaccine production.

### Gene variation

The gene variation of the CTNCEC25 strain was tracked during the adaptation process by sequencing the virus structural protein genes N, P, M, G and L (Table 1). A comparison of the genome sequences of the five structural protein genes among strains at different passage levels revealed that all five of the structural proteins except protein P had a total of 15 nt changes. These mutations resulted in eight amino acid substitutions (genome [nt] positions 2792, 3068, 3812, 4371, 4538, 4635, 4826, and 10212), with one in protein L (aa 1602), two in protein M (aa 99 and 191) and six in mature G protein (aa 147, 333, 389, 421 and 485), respectively. All of these mutations occurred before 30 passages, and the viral genome maintained its stability from 30 to 55 passages in CECs (Table 1).

**Table 1:** Sequence comparison of structural proteins among strains at different passage levels

**Genome position**	**Protein position**	**CTN-1 nt/aa**	**1 nt/aa**	**5 nt/aa**	**6 nt/aa**	**10 nt/aa**	**25 nt/aa**	**30 nt/aa**	**33 nt/aa**	**45 nt/aa**	**55 nt/aa**
461	N-130	G/Arg	A/-	A/-	A/-	A/-	A/-	A/-	A/-	A/-	A/-
2792	M-99	T/Leu		G/Arg	G/Arg	G/Arg	G/Arg	G/Arg	G/Arg	G/Arg	G/Arg
3068	M-191	C/Ser						T/Leu	T/Leu	T/Leu	T/Leu
3812	G-147	A/Lys						G/Glu	G/Glu	G/Glu	G/Glu
4371	G-333	G/Arg			A/Gln	A/Gln	A/Gln	A/Gln	A/Gln	A/Gln	A/Gln
4538	G-389	G/Glu	A/Lys	A/Lys	A/Lys	A/Lys	A/Lys	A/Lys	A/Lys	A/Lys	A/Lys
4635	G421	C/Pro						A/Gln	A/Gln	A/Gln	A/Gln
4636		A/Pro	G/-	G/-	G/-	G/-	G/-	G	G	G	G
4826	G-485	T/Ser	C/Pro	C/Pro	C/Pro	C/Pro	C/Pro	C/Pro	C/Pro	C/Pro	C/Pro
6289	L-294	A/Ser				G/-	G/-	G/-	G/-	G/-	G/-
7078	L-557	G/Leu					A/-	A/-	A/-	A/-	A/-
7750	L-781	G/Glu	A/-	A/-	A/-	A/-	A/-	A/-	A/-	A/-	A/-
9886	L-1493	G/Pro						T/-	T/-	T/-	T/-
10141	L-1578	A/Leu					G/-	G/-	G/-	G/-	G/-
10212	L-1602	G/Arg						A/Lys	A/Lys	A/Lys	A/Lys

The CTNCEC25 strain’s genomic stability from 30 to 55 passages was further confirmed by whole genome sequencing at the 33, 45 and 55 passage levels. A comparison among these three passage strains revealed a 100% shared identity at the whole genome nucleotide level (unpublished data). The genomes of the CEC cell culture-adapted virus contain 11,924 nt, with 17 changes compared with the parental CTN-1 V and the nucleotide sequences of these two viruses were 99.9% identical (unpublished data). In addition to the mutations in the structural protein-coding regions mentioned above, there was one deletion in the poly A tail of the P gene, and there was one nucleotide substitution located at the 3’ untranslated region of the G gene. No changes were observed in any other part of the CTNCEC25 viral genome.

## Discussion

In this study, we screened CEC-adapted RABV strain CTNCEC25 from the CNT-1 V strain through serial passages in CECs. The virus titer change indicated that the CTN-1 V strain gradually adapted to propagation in CECs following the passages. During this adaptation process, some of the viral characteristics changed. The most prominent was that the passage of the virus in CECs reduces its pathogenicity in adult mice. In adult mice, the CTNCEC25 strain was totally nonpathogenic at the highest tested dose (as high as 10^7.9^ FFU/ml) after passage level 20. Conversely, the immunogenicity of the CTNCEC25 strain increased during the mouse immunization test along with the passage series in CECs, and the protection index reached up to 120,000 at passage 45, which was much higher than that of the parental CTN-1 V strain and the standard for the Pharmacopoeia of the People’s Republic of China (2010), Volume III. The NIH potency of inactivated vaccines without further concentration and based on the CTNCEC25 strain reached as high as 6.65 IU/ml, indicating that the immunogenicity of the CTNCEC25 strain is sufficient to permit inactivated vaccine production.

The G protein is the most relevant component of the RABV because of its multiple functions in the RABV replication cycle, such as its attachment to host cells, low pH-dependent membrane fusion, viral virulence [18-20] and for eliciting the production of neutralizing antibodies, etc. [21]. Previous studies have demonstrated that the presence of Lys/Arg-333 in mature G proteins in RABV is essential for the lethality of the RABV strain in adult mice [18,22,23]. Our results indicated the Arg-333 in the CTN-1 V strain was mutated to Gln-333 during serial passages in CECs (Table 1). Accordingly, the pathogenicity of the CTNCEC25 strain also decreased greatly and totally lost viral virulence in adult mice totally after passage level 20 (Figure 1). However, it was noted that the amino acid mutation at position 333 and the pathogenicity reduction were not synchronous during CTN-1 V adaptation to CECs. The amino acid substitution of Arg-333 to Gln-333 was first detected at passage 6 in CECs, and the virulence of the CTNCEC25 strain started to decrease at passage 11; the strain became totally apathogenic to adult mice after passage 20. The delayed effect of mutating Arg-333 on the virulence of CTNCEC25 may be explained from two aspects. First, the Gln-333 substitution strain required natural screening through several passages to be purified because the gene mutations of all viral particles may not occur at the same time. Second, viral reproduction was improving more and more in CECs following the serial passages, which could be verified by the increasing virus titers from passage 6 to 10 (Figure 1). Therefore, the impact of increasing the virus titer may offset some of the pathogenicity reduction effects of the virus solution.

The mutation of Lys-147 to Glu-147, which is another amino acid substitutions observed in the CTNCEC25 strain, may affect the function of the virus. Previous studies have implicated the mutations of Lys-147 to Gln-147 mutation in mature G proteins in RABV for reducing its pathogenicity after i.m. inoculation of the virus into adult mice. Christophe Prehaud et al. 1988 also found that mutations in position aa 147 conferred partial or total resistance to most MAbs that recognized antigenic site II, suggesting this amino acid may be associated with the viral immunogenicity [24-28]. In our study, the CTNCEC25 strain had amino acid substitutions at position aa 147 in passage 30. However, the direct pathogenicity reduction of CTNCEC25 at passage 30 for adult mice could not be detected because the virus already had lost its pathogenicity for adult mice at passage 20, which could have occurred possibly because of a Gln-333 substitution. However, the immunogenicity effect of the mutation was obvious. The protection index increased to 4,266 at passage 30 from 600 at passage 25, and it drastically increased up to more than 10,000 after passage 33.

Likewise, amino acids at positions 34, 164, 182, 198, 200, 205, 210, 242, 255, 268, and 303 of mature G protein have been found to be associated with the pathogenicity of RABV strains in adult mice [22,24,29,30]. However, none of these virulence-associated aa residues were changed during the cell culture adaptation of the CTN-1 V strain into the CTNCEC25 strain.

In addition to the two RABV pathogenicity-associated amino acids mutations mentioned above, there were five other amino acid substitutions in proteins G, L and M in the CTNCEC25 strain. Following passage in the CECs, these five amino acids were gradually changed. Among these changes, the amino acid substitutions at positions 389 and 485 of the G protein reverted to the same ones found at the corresponding genome positions in some other cell culture-adapted strains, such as strain PV and SAG2, suggesting that these changes might be involved in adapting to cell culture. Amino acid 1602 of the L protein may not be conserved because the mutation was found at this position in some other cell culture-adapted strain and street isolates. Two other aa site substitutions, namely aa 421 of the G protein and aa 99 of the M protein, were not found at the same genome positions in street rabies virus isolates and other vaccine strains, and their function requires further study.

In conclusion, we have successfully obtained a CEC-adapted RABV CTNCEC25 strain from the CTN-1 V strain by serial passage in CECs. The new adapted strain CTNCEC25 lost virulence in adult mice but retains its high immunogenicity and high propagation rate in cultured cells, which make it an ideal candidate for inactivated human vaccine production.

## Materials and methods

### Cell line and virus strain

Vero cells were obtained from the America Center for Type Culture Collection (ATCC, CCL-81). BSR cells were a gift from Professor Tangtsing of the Chinese Centre for Disease Control and Prevention (CDC, P.R. China). The RABV strain CTN-1 V, the CVS and an aG adapted to the BSR cell line were provided by the National Institute for Food and Drug Control, (NIFDC, P.R. China). SPF eggs were supplied by Guangdong DaHuaNong Animal Health Products Co., Ltd (Xinxin County, Guangdong Province, P.R. China) and they were incubated for 9 days to prepare the primary CECs.

### Culture medium

Vero and BSR cells were grown in Dulbecco’s modified Eagle’s medium (DMEM; Invitrogen, Glasgow, UK) supplemented with fetal calf serum (FCS; 3–10%; Gibco Invitrogen cell culture, Glasgow, UK). Primary CECs were maintained in Medium 199 (M199, Invitrogen, Glasgow, UK) supplemented with 10% FCS and 1% human albumin (Shenzhen Weiguang Biological Products Co., Ltd, Shenzhen, P.R. China). Tryptose phosphate was supplied by Invitrogen (Glasgow, UK).

### Virus titration

The virus titer was determined using a modified rapid fluorescence focus inhibition test as previously described [31] and expressed in fluorescent focus units (FFU)/ml. Briefly, a monolayer of BSR cells in 96-well plates was incubated with serial three-fold virus dilutions. At 24 h post-infection (pi), the cells were fixed with 80% ice-cold acetone and stained with a FITC-labeled N-protein-specific monoclonal antibody (Millipore). The plates were examined by fluorescence microscopy (Olympus Corp., Tokyo, Japan), and the number of fluorescent foci presented in the wells was recorded. Endpoints were defined as the highest dilutions with fluorescent foci less than 30, and virus titers were calculated by the following formula: virus titer (FFU/ml) = (the mean foci number in the endpoint wells × 5 + the mean foci number in the wells with lower dilutions next to the endpoint well) ÷ 2 × the dilution factor of the lower dilutions × 20.

### Virus propagation and passage in primary CECs

The CEC-adapted CTN-1 V strain CTNCEC25 was prepared as follows. Firstly, the CTN-1 V strain was propagated in Vero cells for 10 passages. For each passage, the virus was cultivated on monolayer of Vero cells in DMEM supplemented with newborn calf serum at 35 - 37°C in a 5% CO_2_ humidified incubator at a 10^-2^- 10^-3^ dilution for 4–6 days, and the virus-containing supernatant was then transferred to the second passage of Vero cells in the same dilution. Then, the virus-containing supernatant was transferred to 9-11-day-old chick embryos at a dilution of 10^-1^ - 10^-2^, and were incubated at 35 – 37°C for another 7 days. Finally, the primary CECs were infected with the virus produced in chick embryos at a cell concentration of 1 × 10^6^ cells/ml with a multiplicity of infection (MOI) of approximately 0.001 - 0.05 FFU/cell. Virus propagation was performed at 33-35°C in 25-cm^2^ Kolle flasks containing 5 ml of M199 supplemented with human serum albumin and newborn calf serum for 4–5 days of incubation. This step was continued and optimized until an acceptable virus titer was reached (≥10^7^ FFU/ml). The resulting CEC-adapted CTN-1 V strain was named CTNCEC25 (GenBank accession no. KJ466147).

### Mouse Inoculation Test (MIT)

Viral virulence in adult mice was measured in 4-week-old Kunming mice. Groups of five adult mice were intracerebrally (i.c.) inoculated with 0.03 ml of serial 10-fold dilutions of each virus. Clinical disease signs and death numbers were observed and scored daily for 14 days. Any death occurring during the first three days was discarded as a nonspecific death. The median lethal dose (LD_50_) of each virus was calculated using the Reed and Muench method [32].

### Pathogenicity assay

A pathogenicity study of the viruses was performed using an in vivo infection model as previously described [33]. A group of ten two-day-old Kunming suckling mice was inoculated i.c. with 0.03 ml, or ten adult mice of different sizes were inoculated i.c. with 0.03 ml, intramuscularly (i.m.) with 0.1 ml, intraperitoneally (i.p.) with 0.5 ml or intradermally (i.d.) with 0.1 ml of the CTNCEC25 (10^7.91^ FFU/ml) and CTN-1 V (10^7.98^ FFU/ml) strains, respectively. Clinical disease signs and death number were observed and scored daily for 21 days. Any death occurring during the first three days was recorded, any dead mouse was then discarded. The production of anti-rabies antibodies in surviving mice was evaluated at the end of the experiment.

### Preparation of the experimental vaccine

Test vaccines were prepared from seed material adapted to primary CECs in accordance with the virus production process in primary CECs as specified above. The harvested viruses were first clarified by filtration through a 0.65-μm filter and then inactivated using β-propiolactone. The vaccine was then evaluated using an NIH test and a mouse protection test by comparing the vaccine with a standard vaccine.

### Immunogenicity studies

Vaccine candidates were injected i.p. into adult mice (12–14 g, 0.5 ml; 1 dilution per group) on days 0 and 7. Mice injected with PBS instead of vaccines were used as negative controls. Fourteen days after the first immunization, both the experimental and control groups were challenged with i.c. injections in serial ten-fold dilutions of standardized virus (CVS strain, 0.03 ml, 10 mice per dose). The numbers of mice dying of rabies between 4 and 14 days post-challenge were recorded, and the protection index was determined as the ratio of the LD_50_ of the sample group to that of the control group. Serum samples were collected from surviving animals for an indirect fluorescent antibody (IFA) test, as previously described [34], for the anti-rabies antibodies at the end of the experimental period (days 7, 14, 21, 28 and 42).

### Vaccine potency test

Three passage (passage 36, 40 and 45) viruses were selected to produce vaccines without concentration. The potency of the prepared vaccine was determined according to the NIH test [35]. Groups of 16 4–6 week-old Kunming mice were given two 0.5 ml doses of different vaccine dilutions i.p. on days 0 and 7. The immunization was followed by an i.c. dose of the challenge virus standard (CVS) strain (0.03 ml, containing 32 LD_50_) dose 14 days after the first vaccine inoculation. A national reference calibrated to the international reference vaccine and a titration of the challenge virus was included in each test series. The median effective dose (ED_50_) value of the vaccine under examination was calculated and compared with the ED_50_ of the national reference preparation, and the relative potency of the vaccine was calculated and expressed in International Units (IU/ml). In addition, the anti-rabies antibodies of mice immunized with vaccine prepared from passage 36 were detected using IFA tests at days 3, 7, 10, 14, 21, 28 and 42 pi.

### DNA sequencing

Genomic RNA was extracted from the viruses at different passage levels with a QIAamp Ultrasens Virus Kit (QIAGEN) according to the manufacturer’s instructions. Full-length cDNA was synthesized using First Strand cDNA Synthesis Kit-Rever Tra Ace-α reverse primer (Toyobo Life Science, Shanghai, China). According to the CTN-1 genome sequence (GenBank accession no. FJ959397), 9 pairs of primers (Table 2) were designed to amplify genes encoding the structural proteins N, P, M, G and L of the virus of some passages from their full-length cDNA sequences. The PCR products were then subjected to nucleotide sequencing by BGI-Shenzhen, Shenzhen, P. R. China. The full-length cDNA of selected passages was directly subjected to nucleotide sequencing by BGI-Beijing, Beijing, China and Invitrogen Life Technologies Corporation, Shanghai, P. R. China.

**Table 2:** Primers used to amplify the CTNCEC25 structural protein gene sequence

**Primer**	**Sequence (5′ → 3′)**	**Target gene**
N-F	ACGCTTAACAACCAAATCAAAG	N
N-R	TTGACGAAGATCTTGCTCAT	
P-F	CGTACTCTAGTGACTCGTAA	P
P-R	ATCTTGCGTAGAAAGTTCAT	
M-F	GGTGGGTTGCTCTGGCTAA	M
M-R	AGGCAGAAGACACCGTTATT	
G-F	ATACGGGCTTAACTCCAACCT	G
G-R	GCTCGGCCTCTGACTCAAT	
L1-F	AGGGTCATATCTTCATGGGA	L
L1-R	TCGCTCGCCAAGCACTCC	
L2-F	TGGAAATTCAGGTTATGAAGTC	
L2-R	ACAGGGCTTTCCTGATCGCATC	
L3-F	CGAGGTAACATCTTGGTGCC	
L3-R	TGAGTCATGTATCGCGACCA	
L4-F	CTCAGGGGCTTCTATATT	
L4-R	ATCATCTCCTCCACTCAT	
L5-F	GTGGGTTTGTTCCGCTC	
L5-R	AGAGGTTCTGATTTGAGA	

### Animal experiment

Kunming mice were supplied by the Medical Experimental Animal Center of Guangdong Province (Guangdong, China). The care and use of laboratory animals was approved by the Animal Care and Use Committee of Weiguang Biological Products Co., Ltd. All animals were treated humanely and euthanized by cervical dislocation (mice) at the end of the experimental period.

## Abbreviations

RABV: Rabies virus; RT-PCR: PCR: Polymerase chain reaction; HDCV: Human diploid cell vaccine; PCEC: Purified chick embryo cell; CECs: Chick embryo cells; FFU: Fluorescent Focus Units; pi: Post-infection; MOI: Multiplicity of infection; LD50: The median lethal dose; ED50: The median effective dose; MIT: Mice Inoculation Test; i.c.: intracerebrally; i.m.: intramuscularly; i.p.: intraperitoneally; i.d.: intradermally; IFA: Indirect fluorescent antibody; IU: International Units.

## Competing interests

The authors declare that they have no competing interests.

## Authors’ contributions

CPG* is the corresponding author and provided overall supervision, participated in designing the study and drafted the manuscript. CHW carried out the design of the study, experimental implementation and the data analysis. SL performed cell culturing and viral passage. SMZ participated in gene sequencing analysis and helped to draft and edit the final manuscript. HL performed PCR analysis and the animal experiment. YDL and LZZ carried out viral titer determination. PZ helped to gene sequencing. XZ participated in designing the study. YJD and WRH participated in project management. KYW helped to culture cells. YPZ participated in early viral passage experiments. WHR and HT performed the animal experiments. All authors have read and approved the final manuscript.

## References

[B1] Knobel DL Cleaveland S Coleman PG Fevre EM Meltzer MI Miranda ME Shaw A Zinsstag J Meslin FX Re-evaluating the burden of rabies in Africa and Asia Bull World Health Organ 2005 83 360 368 15976877 PMC2626230

[B2] Tang Q Li H Epidemic situation and related factors analysis of rabies in China Chin J Epidemiol 2005 26 223 224

[B3] Albertini AA Ruigrok RW Blondel D Rabies virus transcription and replication Adv Virus Res 2011 79 1 22 21601039 10.1016/B978-0-12-387040-7.00001-9

[B4] Mita T Shimizu K Ito N Yamada K Ito Y Sugiyama M Minamoto N Amino acid at position 95 of the matrix protein is a cytopathic determinant of rabies virus Virus Res 2008 137 33 39 18602710 10.1016/j.virusres.2008.05.011

[B5] Wu X Smith TG Rupprecht CE From brain passage to cell adaptation: the road of human rabies vaccine development Expert Rev Vaccines 2011 10 1597 1608 22043958 10.1586/erv.11.140

[B6] Fermi C Über die Immunisierung gegen Wutkrankheit Z Hyg Infekt 1908 58 233 276

[B7] Semple D The preparation of a safe and efficient antirabic vaccine Sci Mem Med Sanit Dept India 1911 1 44

[B8] Vodopija I Clarke HF Baer GM Human vaccination against rabies The Natural History of Rabies 1991 2 FL, USA: CRC Press 571 595

[B9] Pearce JM Louis Pasteur and rabies: a brief note J Neurol Neurosurg Psychiatry 2002 73 82 12082056 10.1136/jnnp.73.1.82 PMC1757303

[B10] Hicks DJ Fooks AR Johnson N Developments in rabies vaccines Clin Exp Immunol 2012 169 199 204 22861358 10.1111/j.1365-2249.2012.04592.x PMC3444995

[B11] Montagnon BJ Polio and rabies vaccines produced in continuous cell lines: a reality for Vero cell line Dev Biol Stand 1989 70 27 47 2759353

[B12] McGarvey PB Hammond J Dienelt MM Hooper DC Fu ZF Dietzschold B Koprowski H Michaels FH Expression of the rabies virus glycoprotein in transgenic tomatoes Biotechnology (N Y) 1995 13 1484 1487 9636308 10.1038/nbt1295-1484

[B13] Yusibov V Hooper DC Spitsin SV Fleysh N Kean RB Mikheeva T Deka D Karasev A Cox S Randall J Koprowski H Expression in plants and immunogenicity of plant virus-based experimental rabies vaccine Vaccine 2002 20 3155 3164 12163267 10.1016/s0264-410x(02)00260-8

[B14] Yarosh OK Wandeler AI Graham FL Campbell JB Prevec L Human adenovirus type 5 vectors expressing rabies glycoprotein Vaccine 1996 14 1257 1264 8961515 10.1016/s0264-410x(96)00012-6

[B15] Jallet C Jacob Y Bahloul C Drings A Desmezieres E Tordo N Perrin P Chimeric lyssavirus glycoproteins with increased immunological potential J Virol 1999 73 225 233 9847325 10.1128/jvi.73.1.225-233.1999 PMC103826

[B16] Lodmell DL Ewalt LC Post-exposure DNA vaccination protects mice against rabies virus Vaccine 2001 19 2468 2473 11257379 10.1016/s0264-410x(00)00475-8

[B17] Singh A Yadav D Rai KM Srivastava M Verma PC Singh PK Tuli R Enhanced expression of rabies virus surface G-protein in Escherichia coli using SUMO fusion Protein J 2012 31 68 74 22134654 10.1007/s10930-011-9373-6 PMC7087916

[B18] Faber M Faber ML Papaneri A Bette M Weihe E Dietzschold B Schnell MJ A single amino acid change in rabies virus glycoprotein increases virus spread and enhances virus pathogenicity J Virol 2005 79 14141 14148 16254349 10.1128/JVI.79.22.14141-14148.2005 PMC1280225

[B19] Faber M Pulmanausahakul R Nagao K Prosniak M Rice AB Koprowski H Schnell MJ Dietzschold B Identification of viral genomic elements responsible for rabies virus neuroinvasiveness Proc Natl Acad Sci USA 2004 101 16328 16332 15520387 10.1073/pnas.0407289101 PMC528969

[B20] Ito H Minamoto N Watanabe T Goto H Rong LT Sugiyama M Kinjo T Mannen K Mifune K Konobe T Yoshida I Takamizawa A A unique mutation of glycoprotein gene of the attenuated RC-HL strain of rabies virus, a seed virus used for production of animal vaccine in Japan Microbiol Immunol 1994 38 479 482 7968680 10.1111/j.1348-0421.1994.tb01812.x

[B21] Wiktor TJ Gyorgy E Schlumberger D Sokol F Koprowski H Antigenic properties of rabies virus components J Immunol 1973 110 269 276 4568184

[B22] Takayama-Ito M Ito N Yamada K Minamoto N Sugiyama M Region at amino acids 164 to 303 of the rabies virus glycoprotein plays an important role in pathogenicity for adult mice J Neurovirol 2004 10 131 135 15204932 10.1080/13550280490279799

[B23] Faber M Li J Kean RB Hooper DC Alugupalli KR Dietzschold B Effective preexposure and postexposure prophylaxis of rabies with a highly attenuated recombinant rabies virus Proc Natl Acad Sci USA 2009 106 11300 11305 19581599 10.1073/pnas.0905640106 PMC2706273

[B24] Prehaud C Coulon P LaFay F Thiers C Flamand A Antigenic site II of the rabies virus glycoprotein: structure and role in viral virulence J Virol 1988 62 1 7 2446011 10.1128/jvi.62.1.1-7.1988 PMC250493

[B25] Benmansour A Leblois H Coulon P Tuffereau C Gaudin Y Flamand A Lafay F Antigenicity of rabies virus glycoprotein J Virol 1991 65 4198 4203 1712859 10.1128/jvi.65.8.4198-4203.1991 PMC248855

[B26] Luo TR Minamoto N Hishida M Yamamoto K Fujise T Hiraga S Ito N Sugiyama M Kinjo T Antigenic and functional analyses of glycoprotein of rabies virus using monoclonal antibodies Microbiol Immunol 1998 42 187 193 9570284 10.1111/j.1348-0421.1998.tb02270.x

[B27] Houimel M Dellagi K Peptide mimotopes of rabies virus glycoprotein with immunogenic activity Vaccine 2009 27 4648 4655 19520204 10.1016/j.vaccine.2009.05.055

[B28] Lafon M Ideler J Wunner WH Investigation of the antigenic structure of rabies virus glycoprotein by monoclonal antibodies Dev Biol Stand 1984 57 219 225 6084612

[B29] Takayama-Ito M Ito N Yamada K Sugiyama M Minamoto N Multiple amino acids in the glycoprotein of rabies virus are responsible for pathogenicity in adult mice Virus Res 2006 115 169 175 16188341 10.1016/j.virusres.2005.08.004

[B30] Yamada K Park CH Noguchi K Kojima D Kubo T Komiya N Matsumoto T Mitui MT Ahmed K Morimoto K Inoue S Nishizono A Serial passage of a street rabies virus in mouse neuroblastoma cells resulted in attenuation: potential role of the additional N-glycosylation of a viral glycoprotein in the reduced pathogenicity of street rabies virus Virus Res 2012 165 34 45 22248643 10.1016/j.virusres.2012.01.002

[B31] Smith JS Yager PA Baer GM A rapid reproducible test for determining rabies neutralizing antibody Bull World Health Organ 1973 48 535 541 4544144 PMC2482941

[B32] Reed LJ Muench H A simple method of estimating fifty per cent endpoints Am J Epidemiol 1938 27 493 497

[B33] Virojanapirom P Khawplod P Sawangvaree A Wacharapluesadee S Hemachudha T Yamada K Morimoto K Nishizono A Molecular analysis of the mutational effects of Thai street rabies virus with increased virulence in mice after passages in the BHK cell line Arch Virol 2012 157 2201 2205 22777181 10.1007/s00705-012-1402-z

[B34] Thomas JB Sikes RK Ricker AS Evaluation of indirect fluorescent antibody technique for detection of rabies antibody in human sera J Immunol 1963 91 721 723 14106292

[B35] Barth R Diderrich G Weinmann E NIH test, a problematic method for testing potency of inactivated rabies vaccine Vaccine 1988 6 369 377 3188617 10.1016/0264-410x(88)90185-5

